# The EPH/Ephrin System in Colorectal Cancer

**DOI:** 10.3390/ijms23052761

**Published:** 2022-03-02

**Authors:** Stavros P. Papadakos, Leonidas Petrogiannopoulos, Alexandros Pergaris, Stamatios Theocharis

**Affiliations:** First Department of Pathology, Medical School, National and Kapodistrian University of Athens, 157 72 Athens, Greece; stavrospapadakos@gmail.com (S.P.P.); lpetrogiannopoulos@gmail.com (L.P.); alexperg@yahoo.com (A.P.)

**Keywords:** EPHs, ephrins, colon cancer, biomarkers, diagnosis, prognosis, therapy

## Abstract

The EPH/ephrin system constitutes a bidirectional signaling pathway comprised of a family of tyrosine kinase receptors in tandem with their plasma membrane-bound ligand (ephrins). Its significance in a wide variety of physiologic and pathologic processes has been recognized during the past decades. In carcinogenesis, EPH/ephrins coordinate a wide spectrum of pathologic processes, such as angiogenesis, vessel infiltration, and metastasis. Despite the recent advances in colorectal cancer (CRC) diagnosis and treatment, it remains a leading cause of death globally, accounting for 9.2% of all cancer deaths. A growing body of literature has been published lately revitalizing our scientific interest towards the role of EPH/ephrins in pathogenesis and the treatment of CRC. The aim of the present review is to present the recent CRC data which might lead to clinical practice changes in the future.

## 1. Introduction

### 1.1. The EPH/Ephrin System

The EPH/ephrin signaling system is part of the broad group of tyrosine kinases, acting as regulators of a wide variety of physiologic processes (axon guidance, angiogenesis, embryologic development of intestinal epithelium). Knowledge of its contribution to carcinogenesis has emerged during the past decade [1,2,3]. Its function has lately been exploited extensively. Gastrulation and somitogenesis are orchestrated by members of the EPH/ephrin signaling pathway, highlighting its fundamental importance for the canonical early morphogenesis [4], while our interest on the normal placental development has been recently renewed due to its substantial contribution [5]. The EPH/ephrin system, given its influence in the lymphocytic maturation in thymus (the EPHB2/ephrin-B1/B2 signaling organizes the thymic medulla [6]) and in the generation of germinal centers [7], has been utilized as a treatment target in a diverse spectrum of inflammatory diseases [8]. In intestinal inflammation, the inhibition of EPHB/ephrin-B forward signaling through EPHB4 or EphB1-Fc in murine models which stimulate the Th1 cells relieves the inflammatory responses [9]. In bone marrow, the EPHB4/ephrin-B2 signaling pathway drives the maturation of erythroid progenitor cells [10] and mobilizes the hematopoietic stem and progenitor cells (HSPCs) into circulation [11]. In parallel, its impact in every step of carcinogenesis has been increasingly documented [1,12,13]. It has even been proposed as a potential treatment target for COVID-19 infection [14].

All the above highlight the concern regarding the orderly operation of this signaling network. Nine EPHAs and five EPHBs binding invariably five glycosylphosphatidylinositol (GPI)-anchored ephrin-A-ligands and three transmembrane ephrin-B ligands, respectively, have been documented. An emphatic characteristic of EPH/ephrin signaling is its bidirectional nature. The onward signaling is mediated by the EPH kinase activity in the EPH-expressing cells while the backward signaling in the ligand-expressing cells is mediated by the Src kinase family [15]. The bidirectional signaling is the principal trigger of the EPH–ephrin complex endocytosis, converting the adhesive interaction into a cell-to-cell repulsive response [16], which is fundamental for many physiologic processes [17]. For example, the abundance and the composition of a cell’s aggregation into ephrin-B1 can influence the transition between epithelial and mesenchymal phenotypes through regulating of the cell–cell junctions [18,19]. Apart from the endocytosis of EPHs or their ephrins, phosphatase-dependent dephosphorylation and peptide cleavage by enzymatic hydrolysis constitute mechanisms that regulate the recycling of EPH components in the plasma membrane [20]. A growing body of literature has also analyzed the contradictory, in a multitude of instances, contribution of EPH–ephrin signaling in colorectal carcinogenesis [21,22], further indicating the immense sophistication of the aforementioned signaling system.

### 1.2. The EPH/Ephrin System in Colon Tissue Formation and Physiology

**Figure 1:**
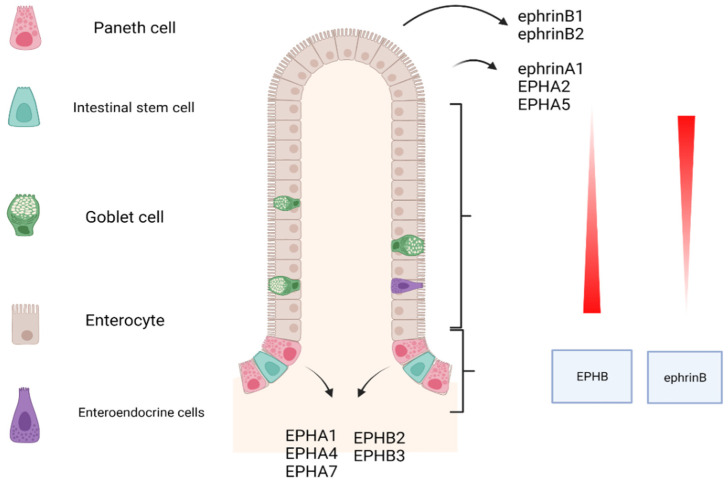
EPH/ephrin expression gradient in gastrointestinal tract. Black arrows: the points of maximal expression. Created with BioRender.com, accessed on 20 February 2022.

The EPH–ephrin signaling system is fundamental for the establishment of the crypt-villous axis and the location arrangement of the cellular populations. The progenitor cells populate the bottleneck of the crypt, stem cells and Paneth cells reside in the bottom of the crypt, and differentiated ones inhabit the villi. The underlying, regulatory mechanism that governs the process is the expression gradient of EPHBs and ephrins in the crypt-villous compartment. Sine qua non, EPHB2 and EPHB3 are maximally expressed in cells that reside in the lowest part of the crypt. Τheir expression declines ascendingly, while the expression of ephrin-B1 and ephrin-B2 has the exact opposite trajectory. It is maximal in the top of the villi and declines towards the crypt [20]. Over and above the effects of EPHB signaling in the gut’s epithelium architecture, EPHBs regulate the proliferation of the intestinal stem cells via the EPHB-kinase-dependent signaling pathway [23]. Analogously, gradients in the gene expression of the EPHA family have been reported. The *EPHA1*, *EPHA4*, and *EPHA7* genes are principally expressed in the crypts while the *EPHA2*, *EPHA5*, and the *EFNA1* coding genes are abundantly expressed on the villi. Nevertheless, the whole spectrum of the implications of this gradient in the structure and the maintenance of the gut epithelium is still lacking [11]. These are illustrated roughly in Figure 1.

### 1.3. The Molecular Pathways of the EPH/Ephrin System

**Figure 2:**
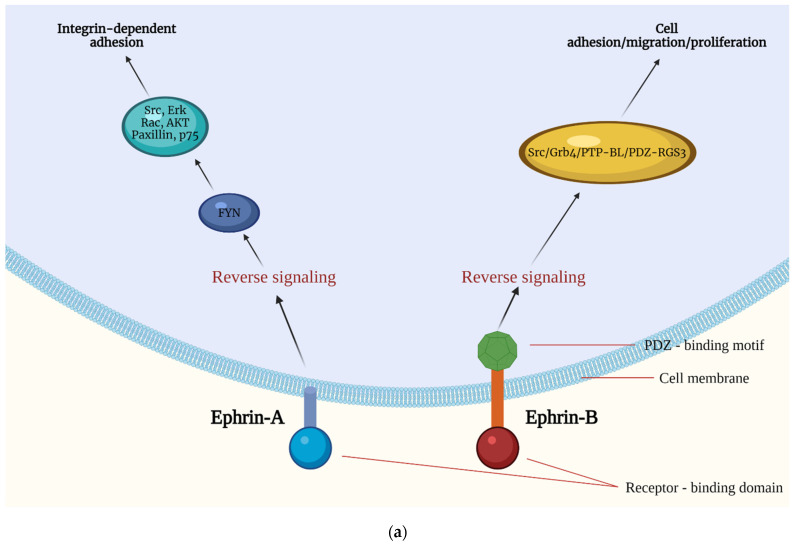

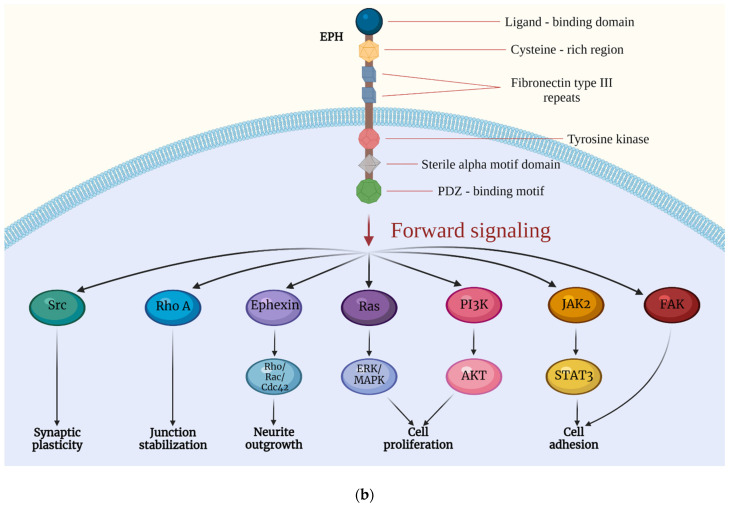
A response is triggered in both the ephrin-expressing cell (**a**) and the EPH-bearing one (**b**). Following EPH–ephrin interaction, an action is triggered in both cells through complex molecular mechanisms, as EPHs/ephrins interact with various molecular cascades to further convey the message into the cytoplasm. Forward signaling includes interaction of EPHs with the Src family kinases, resulting in the regulation of synapses formation, with Rho GTPases leading to junction stabilization as well as interaction with Ephexins and the ERK/MAPK pathway leading to cell proliferation. Moreover, EPHs interact with FAK and the JAK/STAT pathway, resulting in modulation of cell adhesion. Mechanisms implicated in reverse signaling include, among others, interaction between ephrins and Src, Erk, Rac, AΚΤ, paxillin, and p75, leading to integrin-dependent cell adhesion as well as with Src, Grb4, PTP-BL, and PDZ-RGS3, regulating various actions such as cell adhesion, migration, and proliferation. PI3K: Phosphoinositide 3-kinase, JAK: Janus kinase, MAP: mitogen-activated protein, RGS3: regulator of G-protein signaling 3, FAK: focal adhesion kinase, STAT: signal transducer and activator of transcription. Created with BioRender.com, accessed on 20 February 2022.

Activation by a ligand triggers the initiation of the EPH’s intracytoplasmic region RTK activity, which in turn activates molecular cascades that further transmit the signal, a process called forward signaling. GTPases of the Rho and Ras family, focal adhesion kinase (FAK), and the pathways of the Janus kinase (JAK)-signal transducer and activator of transcription (STAT), as well as the phosphoinositide 3-kinase (PI3K), have been observed to participate in forward signaling. EPH–ephrin signaling is bidirectional, as a response is also triggered in the ephrin-expressing cell [24]. The latter process is termed reverse signaling, with proteins such as Src Homology 2 (SH2) or PDZ domain containing proteins, such as Grb4, participating in order to further convey the signal [25,26,27]. The processes of forward and reverse signaling as well as some of the main molecular pathways implicated in them are presented in Figure 2.

### 1.4. Colorectal Carcinoma

Colorectal carcinoma (CRC) is the third most commonly diagnosed cancer in both sexes and, despite the extensive research in diagnostics and treatment, it still constitutes the second most fatal malignancy. An epidemiologic dichotomy among developed and transitioning countries with an approximately ten-fold variance in their incidences is documented. Well-recognized risk factors include, among others, the shift towards a more sedentary way of living with the consequent rising wave of obesity and a diet enriched in animal proteins and fats and deprived of whole grain and fibers [28]. Τhe contribution of the genetic component in the development of disease is also largely established. In fact, the role of EPH/ephrin genetics has begun to unfold, with limited results until now. Although four variants of the *EPHB2* gene (I361V, R568W, D861N, R80H) have been reported in Finnish and British populations, none of them conferred a predisposition to developing CRC [29]. In the same direction, the rs9520090, which constitutes a single-nucleotide polymorphism (SNP) of the *EPHB2* gene, seems to affect the OS rather than affecting the development of CRC due to its role in angiogenesis [30]. Finally, the mutation burden of the EPHs occur mainly at late stages of the disease, concerning principally fibronectin type III and the tyrosine kinase [31].

## 2. Molecular and Biological Relevance of EPH/Ephrin in CRC Models

The EPHA2-ephrin-A1 interplay is of critical importance for eschewing contact-inhibition. In spheroid cultures of HT-29 cells, in which the maximal cell–cell contact is applied, the downregulation of ephrin-A1 resulted in a mitigation of growth rate, interfering with the tyrosine phosphorylation of EPHA2, E-cadherin, and b-catenin, as indicated by their hyperphosphorylation under the influence of EFNA1-Fc [32]. Eriksson O. et al. demonstrated that EPHA2 and tissue factor (TF) are synchronously expressed in CRC surgical specimens. In the MDA-MB-231 breast cancer cell line, the factor VII (FVII) which is a ligand of tissue factor (TF), was shown to enhance the ephrin-A1-mediated activation of the RhoA/ROCK signaling pathway irrespective of PAR-2, inducing a “mesenchymal-to-amoeboid” transformation which could be a metastasis-potentiating event [33]. In the same direction, Li M. et al. documented, in EPHA3-overexpressing cells grafted into nude mice models, the upregulation of expression of certain mRNAs and lncRNAs which are implicated in cytosolic DNA-scavenging in MAPK, VEGF, and transcription signaling pathways as well as in apoptosis and in the downregulation of specific genes that are involved in the metabolism of the conditionally essential amino acids arginine and proline, apoptosis, and cytoskeleton rearrangement [34]. Collectively, according to Li M. et al., EPHA3 regulates several signaling pathways contributing to CRC development. These results are contradictory with the documentation of Andretta E. et al. According to the latter, the overexpression of wild-type EPHA3 in LS174T and DLD1 colon cancer cell lines in parallel with the overexpression of ephrin-A5, its preferred ligand, did not have any influence on cell growth and motility or on their metastatic capacity in vivo. Analogously, the upregulation and the downregulation of mutant EPHA3 expression did not exhibit any impact on cell growth and motility, while silencing of EPHA3 neither triggered carcinogenesis nor altered the tumor size of established intestinal tumors. Overall, they downplayed the contribution of EPHA3 signaling in colorectal carcinogenesis [13].

Initial reports concerning the expression of EPHB2, EPHB3, EPHB4, and ephrin-B2 mRNA document significant alterations in the expression patterns among cancerous colonic cancer cells lines, surgical specimens, and the contiguous healthy tissue, with ephrin-B2 being the most variably expressed [35]. According to epigenetic studies in twenty-two CRC cell lines, the promoters of the abovementioned EPHs are unmethylated [36]. EPHB2 is activated by the b-catenin/TCF signaling pathway. Larriba M. et al. demonstrated that 1,25(OH)2D3 inactivates the expression of the EPHB2 gene, which can be reversed by Snail1 [37]. Chiou S.T. et al. documented, in the HT-29 cell line, that the upregulation of EPHB3 expression induced growth retardation and apoptosis. The highly EPHB3-expressing cells had an epithelial appearance (tense cell–cell junctions) which became more evident in comparison with the more mesenchymal-like control cells, while confocal microscopy rendered perceptible the accumulation of E-cadherin, F-actin, and b-catenin in the cells’ plasma membrane. The end-result is that EPHB3 signaling promotes tumor-suppressive processes [38]. The Apc^Min/+^ mice have a non-functional Apc (adenomatous polyposis coli) protein due to the existence of the MIN (multiple intestinal neoplasia) allele which encodes a nonsense mutation at codon 850 [39]. These animals are genetically inclined to form tens of adenomas in the small intestine and dysplastic crypts in the colon, accumulating b-catenin in their nucleus and upregulating the expression of the EPHB2, EPHB3, and EPHB4 genes. These render them ideal models to study the initiation and progression of CRC carcinogenesis [39,40]. Battle E. et al. investigated, in Apc^Min/+^ mice, the effects of EPHB2 and EPHB3 expression in CRC by creating mutant receptors lacking their cytoplasmic tail. DcyEPHB2 Apc^Min/+^ mice had ten times the number of tumors in the distal colon with a profoundly more invasive phenotype, as indicated by the desmoplastic reaction, in comparison with the controls. In EphB3^−/−^; Apc^Min/+^ mice, they documented 30% more colorectal tumors, a two-fold increase in tumor size >5 mm in diameter, and a three-fold increase in the percentage of invasive disease in comparison with EphB3^+/−^Apc^Min/+^. The differences among EPHB3^−/−^Apc^Min/+^ and EPHB3^+/+^Apc^Min/+^ more emphatically highlight the contribution of EPHBs’ downregulation in disease progression [40]. Lv J. et al. also investigated the effect of EPHB4 in carcinogenesis in vivo. They established xenograft mice from SW480 CRC cell lines and modified SW480 cell lines to over- (SW480/EPHB4) and under-express (SW480/shEPHB4) the EPHB4. The SW480/EPHB4 mice had an increased growth rate and exhibited a vascular- and muscle-invasive phenotype in comparison with SW480/shEPHB4 and the controls, which indicates that EPHB4 might contribute to tumor progression and metastatic spread [41]. Still, the intensification of vascular density in conjunction with the noteworthy downregulation of E-cadherin expression in tight junctions are suggestive of the abovementioned hypothesis [41].

Taking it a step further, Yekkala K. et al. explored the contribution of c-myc signaling in CRC carcinogenesis utilizing Apc^Min/+^ mice. They reported that Apc^Min/+^ c-myc^+/−^ mice had less small intestine tumors and colon polyps with a smaller diameter than Apc^Min/+^ c-myc^+/+^ mice, which accounted for their OS time. VEGF, EPHA2, and ephrin-B2 had a significant downregulation of their expression correlating with the c-myc expression levels in the small intestinal tumors [42]. Dominguez-Brauer C. et al. further investigated the role of c-myc by studying Mule, an E3 ubiquitin ligase. They documented that the knockout of Mule in APC^Min^ mice resulted in a nearly ten-fold increase in the number of small and large intestinal adenomas populated by cells from the bottom of the crypts due to Wnt signaling hyperactivation. Summarizing a series of more sophisticated experiments, they suggested that the loss of Mule, which orchestrates the lysosomal and proteosomal degradation of various molecules, leads to the ectopic overexpression of EPHB3 and the aberrant c-myc-mediated expression of Paneth cells and stem cells. The end result constituted the formation of the abovementioned adenoma [43].

Εphrin-B1 and ephrin-B2 are preferentially incorporated in CRC-derived exosomes [44] while only ephrin-B2 is variably expressed among exosomes derived from SW480 (primary site) and SW620 (LN metastasis) cell lines [45]. Such results indicate that the exosomal ephrin-B1 and ephrin-B2 could serve as diagnostic biomarkers. The loss of ephrin-B1 expression leads to the formation of villous adenomas. Cdx1 and Cdx2 are transcription factors necessary for the intestinal epithelium homeostasis. In human CRC specimens, the downregulation of Cdx2 expression is indicative of more aggressive disease, while Cdx1:Cdx2:APC^Min^ mice are inclined to develop highly invasive, villus neoplasms with reduced ephrin-B1 expression [46]. Ζhu Y. et al. reported that Cdx-2 promotes Notch signaling by binding in the promoter of the Dll1 gene and upregulating the ephrin-B1 expression on the adjacent cells [47]. As mentioned above, ephrin-B2 is aberrantly expressed in CRC tissues compared with the bordering normal ones [35]. Liu W. et al., taking it a step further, explored the effects of ephrin-B2 overexpression through transfection of the KM12L4 colon cancer cell line. The ephrin-B2-overexpressing mice had a significantly shrunken tumor volume, comprising approximately 25% of the tumor volume of the control mice on day 15. Further exploration with 51Cr-labelled RBCs demonstrated a decrease in the tumor blood volume, despite the generation of a tumor vascular network, due to morphologic abnormalities (e.g., thin-walled vessels). Apoptosis, along with the expression of cell cycle regulatory proteins, remained unaltered [48]. Furthermore, ephrin-B2 is linked with the generation of drug resistance in gain-of-function mutant p53 disease. Alan S.K. et al. documented that ephrin-B2 is being upregulated in response to DNA damage by recruitment of the mutated p53. Drug resistance can be attributed to a multitude of underlying molecular mechanisms. They have demonstrated that there is a JNK-mediated expression of the ATP-binding cassette transporter (ABCG2) after the activation of ephrin-B2 reverse signaling. Apart from this, ephrin-B2′s reverse signaling after 5-fluorouracil treatment potentiates carcinogenesis and EMT by promoting the Src-ERK and the Src-FAK signaling pathways, respectively [49]. The data regarding the role of the EPH/ephrin system in colorectal carcinogenesis, as reported from studies that incorporated cell lines and xenografts, are presented in Table 1.

## 3. Prognostic and Predictive Role of the EPH/Ephrin System in CRC

The contribution of EPH/ephrin signaling in the developmental process of embryonic vasculature is well-established in the literature, constituting the foundations for the conceptualization of EPH/ephrin involvement in tumor neovascularization. Oqawa K. et al., experimenting with xenografts from human-derived breast cancer and Kaposi’s sarcoma cell lines, documented the expression of ephrin-A1 and EPHA2 all over the endothelial lining and cancer cells, demonstrating analogous results in surgically resected CRC specimens [51]. It is common knowledge now that certain EPHAs (EPHA1, EPHA2, EPHA8) and EPHBs (EPHB2, EPHB4) are variably expressed in CRC [52,53,54] specimens, compared with the adjacent normal intestinal tissues, with their expression further being downregulated with CRC progression [54].

The downregulation of EPHA1 is suggestive of lower overall survival (OS) [55], in accordance with the poorer progression-free survival (PFS) in patients with elevated EPHA2 expression [56]. Τhe expression of EPHA2 also heralds a weak response in anti-EGFR therapy [56,57,58]. Li M. et al. reported a statistically significant association between EPHA3 expression and CRC grade of differentiation and lymph node (LN) infiltration, which triggered a series of experiments/studies with cell lines and xenografts [34]. In contrast to the aforementioned data, Andretta E. et al. did not report any association between clinicopathological parameters and EPHA3 expression [13]. The EPHA4 immunohistochemical (IHC) expression was correlated with patients’ age, tumor size, depth of invasion, LN status, and the TNM Classification of Malignant Tumors (TNM) stage. Its increased expression was also indicative of poor prognosis [59].

According to Laiho P. et al., EPHBs exhibit a significant variance in their expression among serrated and typical CRC tissues, possibly contributing to the pathogenesis of disease [57]. EPHB2 expression is associated with improved prognosis and better recurrence-free survival (RFS) and OS, as indicated by a multitude of clinical trials [60,61,62]. Similarly, EPHB3 positive staining was associated with better OS and RFS. Additionally, EPHB3 expression was reported to be higher in CRC tissues compared with the adjacent normal mucosa and its expression was noted as downregulated during the conversion from adenoma to carcinoma [62]. Chiu S.T. et al., analyzing data from patients with advanced CRC, demonstrated a noteworthy downregulation of EPHB3 expression [38], while Ulivi P. et al. demonstrated that EPHB4 was substantially overexpressed in the less-inflammatory, right-sided tumors in comparison with their left-sided counterparts [63]. In accordance with the latter, Lv J.H. et al. reported the upregulation of EPHB4 expression in CRC tissues while ephrin-B2 remained unaltered [41].

Epigenetic mechanisms also contribute to colorectal carcinogenesis; namely, miR-645 downregulating the ephrin-A5 mRNA influences tumor growth and metastasis [64]. Finally, patients who do not respond well to neoadjuvant therapy tend to have higher ephrin-B2 expression than the responders [49].

The abovementioned data are documented in detail in Table 2.

**Figure 3:**
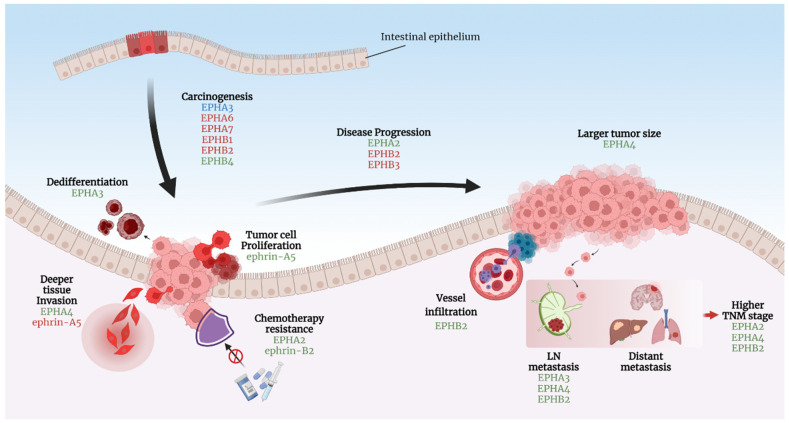
EPHs/ephrins can either enhance or suppress CRC carcinogenesis in various steps. Green fonts: EPHs/ephrins promoting the specific process. Red fonts: EPHs/ephrins inhibit each described action. Blue fonts: Contradictory results have been reported regarding the role of EPHA3 in CRC carcinogenesis. Created with BioRender.com, accessed on 20 February 2022.

The various roles of the EPH/ephrin system in CRC carcinogenesis are presented in Figure 3.

## 4. The EPH/Ephrin System as a Treatment Target in CRC

Although several approaches to therapeutically target the EPH/ephrin system have been developed, currently clinical studies regarding the CRC do not exist. Any detailed narration is beyond the scope of this text and has been done elsewhere [66,67], and only the basic principles will be presented here. Τhe basic therapeutic applications of the EPH/ephrin system include EPH-targeting antibodies, recombinant proteins blocking the EPH–ephrin interaction, peptides carrying chemotherapeutics, and EPH kinase inhibitors [66]. With respect to CRC, the main body of literature consists of preclinical data. Concurrently, the introduction of new systemic therapeutic options such as biologics (anti-EGFR, anti-VEGF drugs), immunotherapy, BRAF-targeting drugs, MEK inhibitors, and salvage agents enriched our therapeutic reservoir while deepening our perception of CRC biology, especially on the differences between right- and left-sided tumors, comprising a valuable source of prognostic and predictive data [68]. Despite those advancements, there are still gaps in our therapeutic approach that could be addressed, and targeting of the EPH/ephrin system could come forward as a therapeutic perspective.

Chemotherapeutics (e.g., capecitabine, oxaliplatin, and irinotecan) have constituted the mainstay of therapeutic options in the systemic treatment of CRC. Their clinical utility has been extended from high-risk stage II disease (e.g., T4 disease, low differentiation) to stage III surgically resected tumors as an adjuvant treatment. In conjunction with biologic agents, chemotherapeutics exhibit notable efficiency in the management of metastatic disease [68]. Their unfavorable side-effects profile, which frequently are additive with therapeutic combinations of fluoropyrimidines with oxaliplatin, comprise a main weakness. The dilemma becomes more challenging when extensive accumulative doses are needed [69]. Towards this direction, attempts to target EPHB2 with monoclonal antibodies (Mab) have been made. 2H9 blocks the interaction between EPHB2 and ephrins, inhibiting the activation of EPHB and impeding the downstream signaling cascade. The conjugation of monomethyl auristatin E(MMAE)with Mab 2H9 restricted the tumor volume of MMAE-vc-2H9-treated xenograft mice (from HT1080-GD and CXF1103 human colon cancer lines), while a four- to ten-fold enhancement in tumor volume was documented in the vehicle-treated mice. This highlights its in vivo clinical effectiveness, which should be further examined in human clinical trials [70]. Formononetin, a natural phytoestrogen extract, showed encouraging results in reducing SW1116 and HCT116 human CRC cell lines growth and migration in a dose-dependent manner. These effects are the end result of a plethora of cellular mechanisms such as the cell cycle arrest in the G0–G1 phase, the downregulation of cyclinD1, the inhibition of matrix metalloproteinase 2(MMP2) and matrix metalloproteinase 9(MMP9), and the upregulation of miR-149. miR-149 causes subsequent cellular growth reduction by EPHB3 downregulation. The principal mediating signaling processes are the inactivation of the PI3K/Akt pathway and the STAT3 phosphorylation [71]. The importance of the EPHB-triggered signaling cascade to support the proliferation of CRC cells has been extensively documented. NVP-Iso, an EPH-specific tyrosine kinase inhibitor (TKI), causes tumor growth retardation on mice models, inducing an autophagy-mediated cell death [72]. The major advantages from the utilization of those agents could be derived by the elective signaling blockage of the EPH tyrosine kinase family, which could significantly limit their side-effects profile. Collectively, the abovementioned agents target distinct cellular pathways and could possibly complement the currently used treatment protocols. 

Another therapeutic contribution might be the enhancement of sensitivity of established therapeutic agents. The introduction of anti-EGFR agents has improved the outcomes of CRC patients. Characteristically, the combination of chemotherapy plus an anti-EGFR agent exerts its superiority over the combination of chemotherapy plus an anti-VEGF. These effects are more emphatic in the left-sided tumors over the right-sided ones, highlighting their biologic differences [73]. The mutation analysis of *RAS* and *RAF* status is of paramount importance, since any downstream mutations can affect the responsiveness to biologics [74,75]. The stimulation of the EPHA2 signaling pathway stimulated the efficacy of cetuximab in patients with NRAS activating mutations and metastatic disease. Metastatic CRC disease with wild-type KRAS status and activating NRAS mutations did not exhibit a clinical benefit from EGFR blocking. Despite the fact that EPHA1 was activated when NRAS^+/+^ cells were exposed to cetuximab and EPHA2 was significantly downregulated, the expression of the EPHA2 gene in NRAS^Q61K/+^cells remained unaffected, indicating a functional relationship among the EPHA2 and EGFR [76]. Furthermore, the drug resistance mechanisms in mutant p53 CRC via ephrin-B2 reverse signaling are thoroughly mentioned above. An approach to target ephrin-B2 as a means to enhance the therapeutic efficacy of DNA-damaging cytotoxic chemotherapeutics could be of therapeutic value [49]. Finally, miR-149 has been documented to act directly on the *TGFB2* gene and regulates cellular proliferation and the sensitivity to 5-fluorouracil (5-FU) [77]. The documentation of miR-149-mimickers’ effectiveness in clinical trials would be of decisive importance, as they could be an invaluable part of our therapeutic artillery against CRC, reducing the toxicity from systemic chemotherapy. 

Metastasis represents a significant cause of mortality in CRC patients. The current therapeutic approach includes the use of chemotherapy in conjunction with biologics [68]. Ephrin-A1 heralds a poor prognosis for CRC patients. Ieguchi K. et al. demonstrated in HEK293 cells that the ADAM-12-cleaved ephrin-A1 (ephrin-A1 174R) phosphorylated the EPHA2 and dephosphorylated Akt comparably with ephrin-A1-Fc while inducing defects in cells’ motility. In xenograft models, the treatment with KB-R7785, which is an ADAM inhibitor, induced an apoptosis-mediated growth retardation and significantly less metastatic burden. The implicated mechanisms were the concomitantly downregulating specific growth factors, such as HB-EGF and IGF [78]. This could revolutionize cancer treatment since it could offer the possibility to prevent metastatic disease. An enormous drawback for their utilization in clinical trials is their poor efficacy and side-effects profile, which are attributed to their broad spectrum of ADAM inhibition. A more targeted approach (e.g., ADAM-12 siRNA or ephrin-A1-targeting antibodies) could attain better clinical results [79]. The implicated therapeutic mechanisms are summarized in Table 3.

## 5. Conclusions

It is evident that EPH/ephrin signaling has an instrumental role in CRC carcinogenesis. Currently, the genetic studies concerning EPH/ephrin signaling remain limited and have failed to demonstrate a predisposing relationship between specific gene variants and CRC [30,31]. EPHA2, EPHA3 and EPHB2, EPHB3 comprise the most well studied predictive and prognostic biomarkers during CRC progression. EPHA2 regulates cellular proliferation and the cytoskeleton and could serve as a prognostic (e.g., PFS, disease progression) and predictive biomarker of the response to cetuximab [56,58]. There are a plethora of clinical specimen data associating EPHB2 and EPHB3 expression with overall survival [43,45], highlighting their potential utilization as prognostic biomarkers. Their clinical significance has been correlated with the in vitro and in vivo analyses. The blockage of EphB2 and EphB3 signaling accelerated the carcinogenesis process [40], indicating their tumor suppressive function. Their probable clinical applicability can be categorized as follows: (a) tumor reductive agents which interact with various components of EPH/ephrin signaling; (b) carriers that target the EPH/ephrin system to deliver cytotoxic agents to tumor cells; (c) enhancers of the efficacy of other biologics or chemotherapeutics; and (d) anti-metastatic agents.

It is becoming evident that therapeutic interventions in the EPH/ephrin system could reshape the architecture of colorectal carcinoma treatment and their efficiency should be validated in clinical trials in humans. Further research could enlighten our unaddressed inquiries towards the molecular mechanisms that govern the EPH/ephrin signaling system in CRC carcinogenesis.

## Figures and Tables

**Table 1:** EPHs/ephrins studied in cell lines and xenografts, methods utilized, and results conducted.

EPHs/Ephrins	Cell Lines/Xenografts	Methods	Outcomes	Refs.
**EPHA2**	HT-29 cell line	Immunoblotting, spheroid cultures	Ephrin-A1 influences EPHA2 tyrosine phosphorylation: Reduced ephrin-A1 mitigates cell proliferation	[50]
	MB-231 breast cancer cell line	Proximity ligation assay, confocal microscopy	Coexpression of EPHA2 and tissue factor: FVIIa potentiates the ephrin-A1-mediated, PAR-2-independent RhoA/ROCK activation	[33]
	Apc^Min/+^ c-myc^+/−^ mice, Apc^Min/+^ c-myc^+/+^ mice	Cell proliferation analysis, RT-PCR, cell death detection in situ	VEGF/EPHA2/ephrin-B2 pathway downregulation: Less small intestine tumors and colon polyps with smaller diameter in Apc^Min/+^ c-myc^+/−^ mice Better OS (of mice)	[42]
**EPHA3**	IMCE-neo, EPHA3-T37K cells/nude mouse xenograft model	IHC, cell cultures, RT-PCR, Western blot	EPHA3 potentiates carcinogenesis	[34]
	LS174T, DLD1 colon cancer cell lines/NOD/SCID, Apc^Min/+^ mice	RNA extraction, RT-PCR, Western blot, IHC	EPHA3 has no influence on tumor initiation, proliferation, and metastasis	[13]
**EPHB2**	13 MSS and 9 MSI cell lines	MSP, RNA extraction, sqRT-PCR, Northern blot, cell cultures	Rarely promoter hypermethylation	[36]
	SW480 cells	Western blot, IHC, RT-PCR, cell cultures	1,25(OH)2D3 downregulates the expression of EPHB2	[37]
	DcyEPHB2; Apc^Min/+^ mice	Cell culture, Northern blot, in situ hybridization, mice	b-Catenin/Tcf4 complex target-Wnt signaling: More distal colon tumors with more invasive phenotype	[40]
**EPHB3**	13 MSS and 9 MSI cell lines	MSP, RNA extraction, sqRT-PCR, Northern blot, cell cultures	Rarely promoter hypermethylation, with Snail1 repressing this effect	[36]
	EPHB3^−/−^; Apc^Min/+^ mice	Cell culture, Northern blot, in situ hybridization, mice	b-Catenin/Tcf4 complex signaling More colorectal tumors with increased tumor size and more invasive disease	[40]
	HT-29 cell line	Cell culture, transwell migration assay, cell aggregationassay, apoptosis detection assay	EPHB3 overexpression induced growth retardation and apoptosis an epithelial phenotype	[38]
	Mule knockout APC^Min^ mice	Whole-exome sequencing, organoids, RT-PCR, immunoblotting	Mule exerts immunosupressive functions	[43]
**EPHB4**	13 MSS and 9 MSI cell lines	MSP, RNA extraction, sqRT-PCR, Northern blot, cell cultures	Rarely promoter hypermethylation	[36]
	SW480 colon cancer cell lines/female Balb/C athymic mice	Cell cultures, mice, tumor MVD, IHC	EPHB4 enhances tumor growth angiogenesis metastasis	[41]
**ephrin-B1**	LIM1215 human colon tumor cell line	Cell culture, Western blot, immunoaffinity capture	Ephrin-B1 is incorporated in CRC-derived exosomes	[44]
	Cdx1:Cdx2:APC^Min^ mice		Reduced ephrin-B1 expression Cdx2 knockout is responsible for highly invasive, villus neoplasms	[46]
	SW480 cells, Cdx1:Cdx2:APC^Min^ mice	Western blot, IHC, RT-PCR, cell cultures, chromatin immunoprecipitation	Ephrin-B1 gene beingin Notch-regulated: Enhanced generation of polyps in intestinal tract	[47]
**ephrin-B2**	LIM1215 human colon tumor cell line	Cell culture, Western blot, immunoaffinity capture	Ephrin-B2 is incorporated in CRC-derived exosomes	[44]
	Apc^Min/+^ c-myc^+/−^ mice, Apc^Min/+^ c-myc^+/+^ mice	Cell proliferation analysis, RT-PCR, cell death detection in situ	VEGF/EPHA2/ephrin-B2 significantly downregulated Less small intestine tumors and colon polyps with smaller diameter in Apc^Min/+^ c-myc^+/−^ mice Better OS (in mice)	[42]
	SW480 primary, SW620 metastatic human colorectal cancer cell lines	Cell culture, Western blot, immunoaffinity capture	Ephrin-B2 is overexpressed in SW620-derived exosomes	[45]
	KM12L4 colon cancer cell line/ephrin-B2-overexpressing mice	Cell cultures, mice, IHC, Northern blot	Significantly shrunken tumor volume Morphologic abnormalities in tumor vascular network	[48]

**Table 2:** EPHs/ephrins studied in patients’ tissues, methods utilized, and results conducted.

EPHs/Ephrins	Malignant Tissues/Controls	Methods	Outcomes	Refs.
**EPHA1**	125 CRC specimens Controls: 18 controls normal colon tissues	qRT-PCR, IHC	EPHA1 expression downregulation significantly correlated with poorer OS (*p* = 0.02)	[55]
**EPHA2**	82 RAS wild-type tumor specimens	IHC	EPHA2 overexpression linked to ⚬ shorter PFS ⚬ increased rate of disease progression EPHA2 overexpression indicates decreased effectiveness of FOLFIRI plus cetuximab	[56]
	TCGA and GEO datasets	IHC	EPHA2 overexpression associated with worse DFS shorter PFS lower rates of complete or partial disease remission with Cetuximab in patients with KRAS mutations lower disease control rates with Cetuximab in patients with KRAS mutations	[58]
**EPHA3**	153 CRC specimens Controls: 53 matched normal tissues	IHC	Downregulation of EPHA3 expression in CRC	[54]
	68 CRC tissues	IHC	EPHA3 expression Upregulated in CRC specimens Associated with: ⚬ higher age (*p =* 0.015) ⚬ lower tumor differentiation (*p =* 0.001) ⚬ LN metastases (*p =* 0.039)	[34]
	159 Dukes C CRC patients	IHC	Variable EPHA3 expression—no association with clinicopathological parameters	[13]
**EPHA4**	102 CRC samples Controls: adjacent normal tissue	IHC	EPHA4 overexpression associated with age (*p* = 0.027) tumor size (*p* = 0.008) depth of invasion (*p* = 0.004) LN metastasis (*p* = 0.013) TNM stage (*p* = 0.005) poor survival	[59]
**EPHA6**	6 CRC patients Controls: 5 normal individuals	RT-PCR, IHC	EPHA6 expression significantly downregulated in CRC	[52]
**EPHA7**	6 CRC patients Controls: 5 normal individuals	RT-PCR, IHC	EPHA7 expression downregulated in CRC	[52]
	153 CRC specimens Controls: 53 matched normal tissues	IHC	EPHA7 expression downregulated in CRC	[54]
**EPHB1**	6 CRC patients Controls: 5 normal individuals	RT-PCR, IHC	EPHB1 expression downregulated in CRC	[52]
**EPHB2**	37 serrated CRCs, 86 typical CRCs	IHC	EPHB2 expression decreased in serrated CRC	[57]
	345 CRCs, 98 LN CRC metastases, 82 CRC liver metastases Controls: 100 adenomas	IHC	EPHB2 expression downregulated during disease progression	[53]
	370 primary CRCs, 39 CRC metastatic specimens Controls: 28 normal tissues, 148 colorectal adenomas, 342 matched normal mucosa specimens	IHC, in situ hybridization	EPHB2 expression correlated with improved prognosis higher recurrence-free survival higher OS	[65]
	159 CRC specimens	IHC	DFS Hazard ratio 2.24 when IHC ≤ 50% OS Hazard ratio 2.23 when IHC ≤ 50%	[60]
	32 fresh-frozen and 567 paraffin-embedded CRC specimens	RT-PCR, IHC	EPHB2 positivity correlated with LN invasion (*p* < 0.001) venous invasion (*p* = 0.001) TNM stage (*p* < 0.001) MSI (*p* = 0.036) higher OS (*p* = 0.049), improved recurrence-free survival (*p* = 0.015)	[61]
**EPHB3**	610 FFPE CRC specimens	qRT-PCR, IHC	EPHB3 positivity correlates with higher OS (*p* = 0.007) higher recurrence-free survival (*p* < 0.001)	[62]
	36 CRC specimens	IHC	Stage-specific downregulation of EPHB3 expression	[38]
**EPHB4**	60 specimens from patients that received chemotherapy (CT) + bevacizumab (B) and 62 specimens from patients treated with CT alone 51 right-sided and 71 left-sided tumor specimens	IHC	EPHB4 expression elevated in right-sided tumors Better OS, PFS for right-sided tumors with CT+B	[63]
	200 CRC specimens Controls: 50 paired normal mucosa specimens	IHC	Upregulation of EPHB4 expression in CRC tissues	[41]
**ephrin-A5**	28 CRC specimens	RT-PCR, IHC	Decreased ephrin-A5 expression enhances tumor proliferation tumor invasion	[64]
**ephrin-B2**	250 CRC specimens Controls: 50 paired normal mucosa specimens	IHC	Ephrin-B2 expression unaltered between CRC and normal tissues	[41]
	21 CRC specimens	IHC	Ephrin-B2 expression increased in neo-adjuvant treatment non-responders	[49]

**Table 3:** Treatment of CRC through EPH/ephrin signaling.

Drug	Cell Type/Animal model	Mechanisms	Outcomes	Refs.
Ephrin-A1 174R (cleaved ephrin-A1)	HEK293 cells	EPHA2 phosphorylation Akt dephosphorylation	Decreased cell motility	[78]
KB-R7785 (ADAM, MMP inhibitor)	Tumor-bearing C57BL/6 WT mice	Apoptosis induction	Tumor growth retardation Reduction of lung metastases	[78]
Ephrin-A1 mimickers/ cetuximab	NRASQ61K/+ cells	EPHA2-mediated Akt MAPK signaling inhibition	Enhancement of responsiveness to cetuximab treatment	[56]
NVP-BHG712, NVP-Iso	EphB4+ HT-29 cells, EphB2+ Colo205 cells/tumor-bearing mice	Phosphotyrosine-dependent EPH signaling blockage	Autophagy stimulation Decreased cell growth/tumor volume	[72]
MMAE-vc-2H9 (monomethyl auristatin E with Mab 2H9)	HT1080-GD, CXF1103 cell lines/tumor-bearing mice		Decreased cell proliferation/tumor volume inhibition	[70]
Formononetin	SW1116, HCT116 cell lines	Cell cycle arrest in G0–G1 phase, downregulation of cyclinD1, inhibition of MMP2–MMP9, upregulation of miR-149	Reduced cell growth, migration	[71]

## Abbreviations

EPH	erythropoietin-producing human hepatocellular receptors
Ephrin	EPH family receptor interacting proteins
IHC	immunohistochemistry
PCR	polymerase chain reaction
RT-PCR	reverse transcription-polymerase chain reaction
q PCR	quantitative reverse transcription-polymerase chain reaction
LN	lymph nodes
OS	overall survival
DFS	disease-free survival
EMT	epithelial-mesenchymal transition
EGFR	epidermal growth factor receptor
CRC	colorectal carcinoma
RFS	recurrence-free survival
